# Genetic Resistance to Rhabdovirus Infection in Teleost Fish Is Paralleled to the Derived Cell Resistance Status

**DOI:** 10.1371/journal.pone.0033935

**Published:** 2012-04-13

**Authors:** Eloi R. Verrier, Christelle Langevin, Corinne Tohry, Armel Houel, Vincent Ducrocq, Abdenour Benmansour, Edwige Quillet, Pierre Boudinot

**Affiliations:** 1 INRA, Molecular Virology and Immunology, Jouy en Josas, France; 2 INRA, GABI UMR 1313 Animal Genetics and Integrative Biology, Jouy en Josas, France; 3 AgroParisTech, Paris, France; Auburn University, United States of America

## Abstract

Genetic factors of resistance and predisposition to viral diseases explain a significant part of the clinical variability observed within host populations. Predisposition to viral diseases has been associated to MHC haplotypes and T cell immunity, but a growing repertoire of innate/intrinsic factors are implicated in the genetic determinism of the host susceptibility to viruses. In a long-term study of the genetics of host resistance to fish rhabdoviruses, we produced a collection of double-haploid rainbow trout clones showing a wide range of susceptibility to Viral Hemorrhagic Septicemia Virus (VHSV) waterborne infection. The susceptibility of fibroblastic cell lines derived from these clonal fish was fully consistent with the susceptibility of the parental fish clones. The mechanisms determining the host resistance therefore did not associate with specific host immunity, but rather with innate or intrinsic factors. One cell line was resistant to rhabdovirus infection due to the combination of an early interferon IFN induction - that was not observed in the susceptible cells - and of yet unknown factors that hamper the first steps of the viral cycle. The implication of IFN was well consistent with the wide range of resistance of this genetic background to VSHV and IHNV, to the birnavirus IPNV and the orthomyxovirus ISAV. Another cell line was even more refractory to the VHSV infection through different antiviral mechanisms. This collection of clonal fish and isogenic cell lines provides an interesting model to analyze the relative contribution of antiviral pathways to the resistance to different viruses.

## Introduction

Only a fraction of individuals infected by viruses show clinical disease, and epidemiological evidences have established that the host genetic background plays an important role in the susceptibility to infections, explaining a significant part of the clinical variability observed within populations. In human, the interest for the genetic bases of resistance and predisposition to viral infections has dramatically increased during the last years (reviewed in [1]). Monogenic resistance to viruses has been demonstrated, generally involving mutations in receptors such as CCR5 for HIV1 [2]–[4], erythrocyte P antigen for the parvovirus B19 ([5]), or in genes controlling the receptor expression such as the fucosyltransferase (FUT2), which is required for the expression of norovirus ABH co-receptors [6]. In fact, predisposition to viral diseases mirrors the resistance and contributes to the variability of their prevalence within populations. Such predispositions may concern multiple infections when the mutation induces a general immune defect: typical genetic predispositions to multiple infections are due to global primary immunodeficiencies, which are generally rare and usually affect hematopoietic cells like in SCID and XLA [7], [8]. On the contrary, a mutation may enhance the susceptibility to a single virus as in the well-studied example of the skin warts and cancer induced by HPV infection in individuals with a mutated cellular zinc regulator [9]. In fact, single gene based predispositions cover a large continuum between these extreme situations depending on the affected pathway, the range of expression of the mutated gene, and other factors such as the age of the host. Thus, *trim5α* is responsible for the restriction of different retroviruses in non human primates through capsid targeting [10], reviewed in [11]. Host susceptibility to viral infections may also depend on several genes with a higher impact of environmental factors. A traditional distinction is made between single gene based predisposition to rare infections and complex predisposition to common infections within populations [1]. Such complex situations of inheritance are modeled by the so-called “polygenic model of inheritance” [12]. However, in many cases a “major gene” or a “major locus” can be responsible for a significant part of the variability of complex traits including many phenotypes of variable susceptibility to infections. Such major genes (or loci) have been identified for viral diseases using polymorphic markers in genome wide linkage analyses. Thus, several polymorphisms including MHC haplotypes have been recently involved in the host control of HIV-1 by a large-scale study [13]. Overall, the genetic determinism of viral diseases in human is still poorly known.

The basis of genetic resistance and susceptibility to viral infections in domestic animals is not better understood, although the scale of modern farming has led to a situation where herds are affected by a few diseases of which the consequences can be dramatic. Selection of domestic animals usually focused on improving production efficiency while keeping a reasonable general fitness. Mutations that confer strong predisposition to multiple infections have certainly been counter-selected, but many important domestic breeds are very vulnerable to well-adapted viruses. Genome wide analyses have been performed in species where the genetic tools (such as dense genetic maps) are available, in order to understand the basis of resistance to diseases. However, major genes have been identified for only a few infections. For example, a strong association between Marek's disease susceptibility and the chicken MHC has been firmly established [14], [15], but a significant part of the genetic susceptibility to this herpes virus infection is explained by other loci that encode lymphocyte surface antigens of unknown functions. In cows, a *trim* gene related to the TRIM5/TRIM6/TRIM34 group was identified as a restriction factor for different retroviruses [16]. In fish, genetic control of the resistance to viral diseases has been reported in many species. Within domestic salmonids populations, wide ranges of susceptibility have been observed for many viruses including Viral Hemorrhagic Septicemia Virus (VSHV), the Infectious Hematopoietic Necrosis Virus (IHNV), Infectious Salmon Anaemia Virus (ISAV) [17]–[21] in rainbow trout, and for Salmon Pancreatic Disease Virus (SPDV) [22], ISAV [23] and IPNV [24] in Atlantic salmon (*Salmo salar*). In common carp (*Cyprinus carpio* L.), strains more or less susceptible to CyHV-3 have been reported [25], [26], the resistance to the SVCV associated to dropsy was improved by selection [27]. Another example of large intrapopulation variation in resistance to viral diseases has been reported in Atlantic cod (*Gadus morhua*) for the nervous necrosis virus (NNV) [28].

Genome wide analyses have been carried out in some cases, and QTL or major genes for the resistance to several viruses have been identified [29], [30], [16], [31], [32], [33]. In particular, in Atlantic Salmon a major QTL explaining 83% of the genetic variance was identified, which indicates that the resistance to IPNV is in this case almost a monogenic resistance [32]. Even if the mechanisms of resistance or predisposition have not been discovered so far, these results show that, as in mammals and birds, there is a strong genetic control of the resistance to viruses in fish. We previously reported a wide range of variation of susceptibility to the rhabdovirus Viral Hemorrhagic Septicemia Virus (VHSV) between gynogenetic trout clones [21]. Within such clones, fishes are homozygous at every locus and fully histocompatible, constituting a propitious genetic context for studying fish response to pathogens [34], [35]. In particular, it is much easier in this context to distinguish allelic variants of a single gene from duplicated genes that are numerous in salmonids, owing to the whole genome duplication experienced in their early evolution [36], [37].

VHSV is a novirhabdovirus with a single strand RNA genome of negative polarity encoding five structural proteins (N, P, M, G and L) and the non structural NV protein specifically expressed in the novirhabdovirus genus [38]–[41]. The viral glycoprotein (G) is the unique protein expressed at the surface of the viral particle and triggers attachment of the virus to target cells via recognition of cellular receptor, endocytosis and pH-dependent fusion of the viral membrane to the endosomal vesicle. Release of the nucleocapsid initiates transcription/replication of the viral genome catalyzed by the polymerase complex. The neosynthesized RNA genomes will further serve as templates for viral replication or be encapsidated to allow budding of viral neoparticles at the cell surface. VHSV is highly cytopathic and induces the apoptosis of the infected cell, which is mediated by the matrix protein [42].

During the viral cycle, intermediates of replication (ds RNA and 5′-triphosphate RNA) can be recognized by cytoplasmic RNA sensors of the RIG-I (retinoic acid inducible gene –I) family, including in fish a RIG-I protein [43] as well MDA-5 (melanoma differenciation-associated gene 5) and laboratory of genetics and physiology 2 (LGP2) homologs [44]. Activation of these sensors triggers their interaction with the key adaptor Mitochondrial AntiViral Signaling (MAVS) leading to the recruitment of distinct kinases and triggering dedicated signaling pathways [43]. The following phosphorylation of the transcription factors NFkB and IRF3 induces their translocation and the production of IFN [43], [45]. Virus induced fish interferons – named IFNφ – are structurally and functionally very similar to type I IFN [46], [47] and induce many conserved effector genes [48], but they differ from mammalian type I interferons by the presence of introns in their genes [49], [50] and the structure of their receptor [51], [52]. Additionally, IFNφ expression can be induced through TLR (Toll like receptor) pathways [53]. In fish, TLR3 has been reported in endoplasmic reticulum where it binds small size ssRNAs while TLR22 is expressed at the cell membrane and recognizes long dsRNAs [54]. These TLRs recruit TIR containing adaptors (TICAMs) and trigger IRF-dependent IFNφ production. Finally, IFNφ binding to their cognate receptors results in the activation of JAK/STAT canonical pathways and subsequent induction of many interferon stimulated genes (ISG), some of which having a known anti-viral activity like Mx, PKR, ISG15 or Vig-1/viperin. Thus, as in mammals, teleost fish antiviral innate immunity is based on interferons and on a large diversity of ISGs ([48], reviewed in [55]). Teleost fish also mount antigen-specific B and T cells responses against viruses [56], which afford a strong protection based on neutralizing antibodies. However, the relative contribution of intrinsic and adaptive mechanisms in the genetic resistance to VHSV is still poorly understood.

In the present work, we demonstrate that the susceptibility to VHSV infection of fibroblast-like cell lines derived from each fish clone is strictly paralleled to the resistance levels assessed *in vivo* in the parent/birth clones to waterborne infection, indicating that predominant mechanisms involved should be intrinsically active in every cell rather than systemic. We characterized this relationship and we took advantage of the system to show for two highly resistant genetic backgrounds, that different mechanisms of virus restriction are involved.

## Materials and Methods

### Fish and cell lines

Rainbow trout belonging to six homozygous clones (B57, A2, B45, A36, A22 and A3) were used [21]. Clones were established after two successive generations of gynogenetic reproduction and further maintained by within-clone single pair mating using sex reversed (XX) neomales [21]. Every next generation, every breeder (male or female) was checked for homozygosity and isogenicity using allelic variation at 10 polymorphic microsatellite markers. Fish were reared in the INRA experimental facilities (PEIMA, Sizun, for breeders, and IERP, Jouy-en-Josas, for infectious challenges).

Rainbow trout cell lines were derived from the six different fish clones as described for the RTG-2 cell line. After fish have been sacrificed by overexposure to 2-phenoxyethanol diluted 1/1000, ovary was extracted, then trypsinased under constant mild shaking for 2 hours. The supernatant was collected in modified Mac Pherson Stoker Eagle's medium (Eurobio) supplemented with 10% fetal calf serum (FCS), 100 IU.mL^−1^ penicillin and 100 µg.mL^−1^ streptomycin. The cell suspension was centrifuged for 5 minutes at 1000 g, and cells resuspended in culture medium at 20°C in a P24-well plate.

### Ethics Statement

All animals were handled in strict accordance with good animal practice as defined by the European Union guidelines for the handling of laboratory animals (http://ec.europa.eu/environment/chemicals/lab_animals/home_en.htm) and by the Regional Paris South Ethics committee, and all animal work was approved by the Direction of the Veterinary Services of Versailles (authorization number 78-28).

### Cells and fish infections with virus

The strain 07-71 of VHSV (serotype 1) and the strain VR-299 of Infectious Pancreatic Necrosis Virus were used [57], [58]. Cell monolayers were incubated with virus at different MOIs for 1 h at 14°C in medium containing 2% of FCS. The virus suspension was then replaced with fresh media with 2% FCS and cells kept at 14°C. Poly I∶C from Sigma (P9582) was used for IFN induction.

Fish from five of the six clones had previously been tested against the VHS virus [21]. For the purpose of this study, an additional fish clone (B45) was challenged in the same conditions, while control challenges were performed at the same time with the other five fish clones. For waterborne infections, fish were incubated with 10^5^ pfu per ml at 10°C for two hours, then kept in UV treated recirculating water.

### Fin explant cultures

Fin explants were cultured following the protocol previously published in [59]. Two experiments were performed independently, depending on the availability of the fish: B57, A2 and A3 on one hand, and A36, B45 and A22 on the other hand. Fin explants were infected 24 h post sampling at the same virus concentration (depending on the size of the fin, this represented 8.10^3^ pfu per mg of tissue for the first experiment and 2.10^3^ pfu per mg of tissue for the second one). Fins were crushed in culture medium and the viral titer measured 4 days post infection.

### RNA extraction and cDNA preparation

Total RNA was extracted using Trizol (InVitrogen), then purified using the RNeasy mini kit (QIAGEN) according to the manufacturer's instructions, and exposed to DNAse treatment. RNA samples were then checked using Agilent Nano Chips and stored at −80°C.

Reverse transcription was perfomed on 1 µg of total RNA using 125 ng of random hexamer primers (Roche), using the Superscript II Reverse transcriptase kit (Invitrogen) according to the manufacturer's instructions.

### Real Time PCR

The level of gene expression was measured by real time PCR with a Realplex^2^ Mastercycler Instrument (Eppendorf) using Power SYBR® Green PCR Mastermix (Applied Biosystems). Each sample is componed by 5 µL of primers (300 nM each), 5 µL of cDNA (diluted 1/10) and 10 µL of PCR Mastermix. Samples were first incubated for 2 minutes at 50°C and for 10 minutes at 95°C, then subjected to 40 amplification cycles (95°C for 15 and 60°C for 1 minute), followed by 15 seconds at 95°C, 15 seconds at 60°C, 20 minutes from 60°C to 95°C and finally 15 seconds at 95°C, to establish the melting curve of PCR products. Gene expressions were computed according to the ABI Prism 7700 user bulletin (Applied biosystems) and normalized to the beta-actin expression level. Primers used for quantitative PCR (QPCR) are indicated in Table 1.

**Table 1:** qPCR primers sequences.

Gene (Reference for published primers)	Sequence (5′, 3′)
shIFNϕ1 (45)	GCGAAACAAACTGCTATTTACAATGTATA TCACAGCAATGACACACGCTC
lIFNϕ1 (45)	CACGCGAAGTTATTAGCAGTTGAA AAATTATAGTTGAACCACAATGAAATATTATTC
Mx1 (46)	GGTTGTGCCATGCAACGTT GGCTTGGTCAGGATGCCTAAT
Mx3	GATGCTGCACCTCAAGTCCTACTA CGGATCACCATGGGAATCTGA
VHSV-N	CCTGGTGAACAGGTGTCCTT TTCATAGAGGGGGTTTGCAC
gRNA_VHSV_	CAAATTACGGGATTCCGATG TGTGATCATCTCACGGAGGA

### Evaluation of cell monolayer destruction with crystal violet coloration

Cytotoxic effect of viral infection was correlated to cell destruction, evaluated at different times post- infection. Following viral infection and plaque formation, cell monolayers were fixed with 10% Formol for 1 hour at room temperature, then colored with a solution of 1% crystal violet in ethanol for 1 hour at room temperature and washed with tap water.

### Evaluation of cell monolayer destruction with DAPI coloration

Cell monolayers were infected as previously described and fixed after virus absorption or 3 days post-infection in 4% paraformaldehyde. Cell were then permeabilized with 0.2% Triton ×100 before treatment with nuclear marker 4′,6-diamidino-2-phenylindole (SIGMA). Quantification of cell nuclei was then performed from images acquired with 10× objective on a Zeiss Axio Observer-Z.1 microscope by IMAGE J software automatic analysis.

### Plaque assay

Titers of infectious virions were measured by plaque assay on monolayers of EPC cells. Supernatants were collected, and serially diluted in duplicates for the plaque assay. The infection was performed at 14°C under a layer of methylcellulose (0.75% final concentration) for three days after an adsorption step at 14°C for one hour in liquid phase. The plaques were then counted after treatment by formaldehyde (10%) and staining using crystal violet (1% final dilution).

### Western Blot

Clones cells or EPC were transfected with N_VHS cDNA kindly provided by Stephane Biacchesi (INRA, Jouy en Josas). Cells were washed in PBS before lysis in buffer (Tris pH8 50 mM, EDTA 5 mM, MgCl2 15 mM, NP40 1%, NaCl 150 mM) supplemented with a protease inhibitor cocktail (complete EDTA free, Roche). After a short centrifugation (1000 rpm, 5 min.), 50 µg of cell lysate proteins were methanol precipitated for 1 hour at −20°C before centrifugation at 12,000 rpm for 30 min. Pellet were resuspended in sample buffer before analysis by SDS PAGE in 4–12% Nupage gels (Invitrogen) and electrotransfer onto nitrocellulose membranes (Biorad) stained with red ponceau before treatment with anti-N_SHV_ monoclonal antibody (34F5) and secondary anti mouse antibody coupled to horseradish peroxydase. Immunoreactivity was visualized by enhanced chemiluminescence (GE, Healthcare).

### Ranking of trout clones for in vivo susceptibilty to virus (Survival Analysis)

In order to compare accurately the *in vivo* susceptibility of the six fish clones, we combined in a single dataset the results of the previous challenges [21] and those of the additional ones (this study). Thus, data from 9 independant waterborne VHSV experimental infections and a total of 5166 fish were available. The surviving status (dead or alive at the end of the challenge) and the time to death of each fish (in days after infection) were registered. Surviving fish correspond to ‘censored’ observations, *i.e.* that the expected event (death) was not recorded during the observation period. Survival analysis models allow the joint analysis of censored and non censored data. As they take into account both end survival and the kinetics of mortality, they provide an accurate ranking of the susceptibility (relative risk) of groups. The dedicated software named « The Survival Kit » [60] was used to calculate raw (Kaplan-Meier) estimates of the survivor curve S_i_(t) for each clone. A graphical test based on a plot of log(−log S_i_(t)) *vs* log(t) showed that the hazard functions of the various clones were roughly proportional. Using the same software, relative risks were calculated using a Cox regression model [61].

## Results

### Resistant and susceptible fibroblastic cell lines from several trout clones

**Figure 1:**
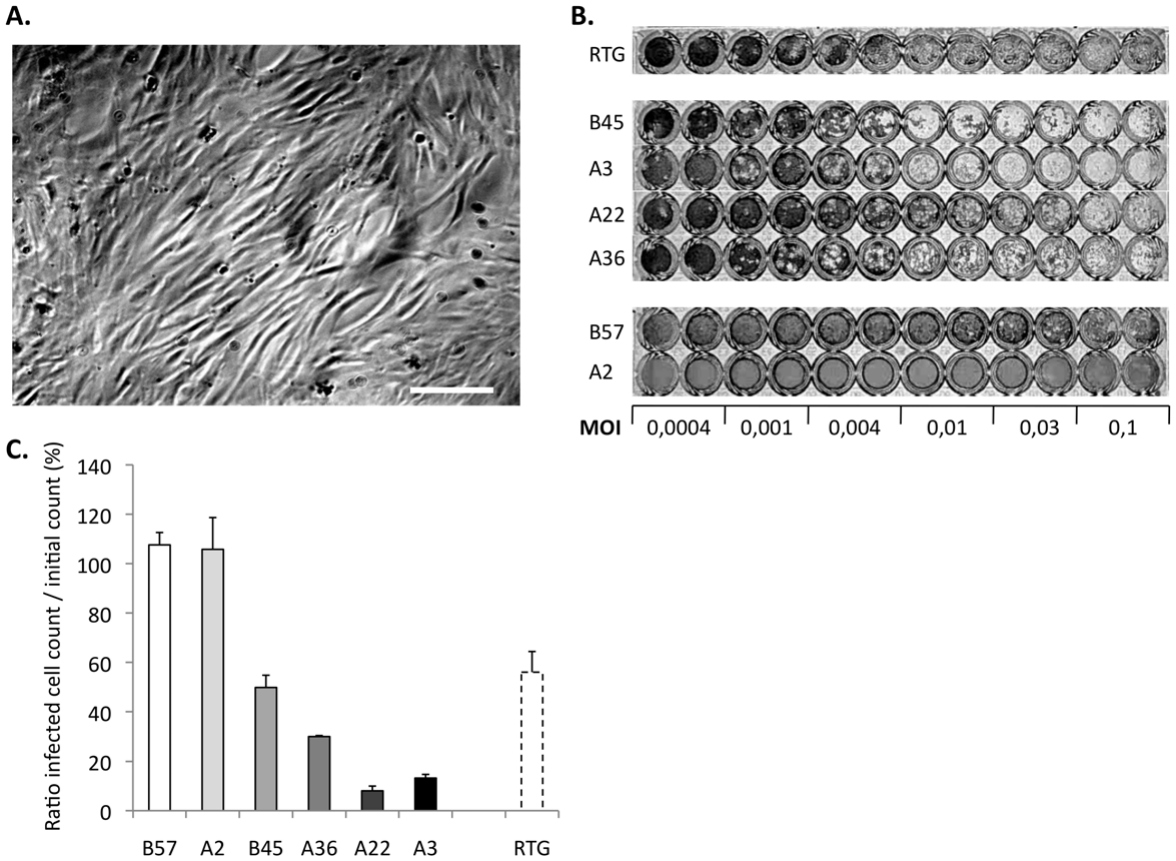
Fibroblastic cell lines from double haploid fish clones show different susceptibilities to VHSV infection. (A). Fibroblast-like cells from the B57 line. Bar 50 µm. (B): Monolayer destruction 3 days post infection with different MOI of VHSV. Cells were incubated 3 days with the virus inoculum, then fixed and colored with crystal violet. (C) Quantification of CPE after VHSV infection (MOI 1): cells were infected as indicated in Materials and Methods, colored with DAPI 3 days post infection, and nuclei counted using the ImageJ software. Three independent infections were performed. Results are shown as ratios of cell counts in infected wells to cell counts before infection. This ratio may be >1 when cell growth occurs after infection in the absence of cytopathic effect.

A collection of homozygous clonal lines of rainbow trout produced by gynogenesis showed a wide range of survival rate to VHSV infection [21]. To establish an *in vitro* system for the analysis of the resistance mechanisms, we derived several cell lines from the ovary of fish homozygous clones. Cell lines were derived from six fish clones, representing susceptible and resistant genetic backgrounds. After 1 month of culture we obtained pseudo-stable cell lines with typical fibroblast morphology (Figure 1A). To assess cell susceptibility to virus infection, we infected the cell lines at different MOIs. The RTG-2 cell line was used as a reference for hyper susceptibility (Figure 1B). In a first analysis, sensitivity to virus infection was assessed by observation of the cytopathic effect (CPE). Amoung the 6 cell lines, we could distinguish three classes of phenotypes: resistant (B57 and A2), susceptible (A36 and A22) or hyper- susceptible (A3 and B45). To better characterize the cytopathic effect (CPE), we evaluated the cell death triggered by virus infection (MOI 1) three days post-infection. While no cell death was recorded in B57 and A2 cell lines upon exposure to the virus, the destruction rate reached 87% and 92% for cell lines A3 and A22, respectively. A36 and B45 cell lines showed intermediary phenotypes with 70% and 50% of destroyed cells after infection (Figure 1C).

Thus, the different cell lines derived from fish clones provided a set of culture systems with a large range of divergent susceptibility to the VHSV infection.

### Early expression of viral N mRNA is correlated to later CPE and viral production

**Figure 2:**
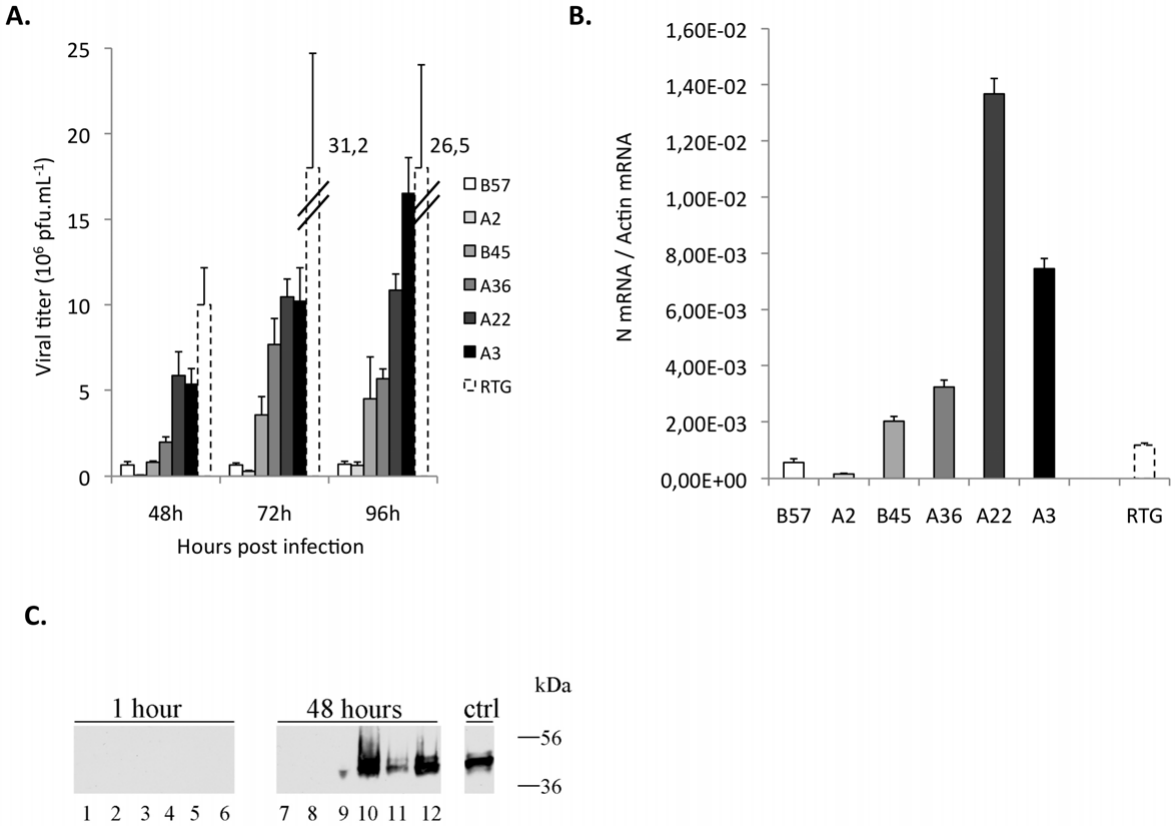
Viral titer, production of N transcripts and protein by the different cell lines. (A) The viral titer in cell supernatant 2, 3 and 4 days post infection with VHSV at MOI 1 was measured by plaque assay using EPC cells. Two independent lines for each genetic background were tested in the experiment, and the plaque assays operated in duplicates. (B) Expression of the N viral transcript 4 hours post infection by VHSV at MOI 1 was measured using qPCR. Transcript copy numbers were normalized to the ß-actin expression (measured ratio of VHSV N mRNA/actin mRNA). Mean values of triplicates are shown. (C) Visualization of the N viral protein by Western Blotting. Infected cell lysates were treated with anti-N_SHV_ monoclonal antibody (34F5). **1 hour**: cells were incubated with VHSV 07-71 during only one hour and lysates prepared for western blotting. (1): B57 (2): A2 (3): B45 (4): A22 (5): A3 (6): RTG. **48 hours**: cells were infected as described in Material and Methods, and lysates prepared 48 hours post infection for western blotting. (7): B57 (8): A2 (9): B45 (10): A22 (11): A3 (12): RTG. **Ctrl**: EPC cells transfected with NSHV cDNA.

Virus production was first evaluated through viral titration experiments in the different cell lines 48 h, 72 h or 96 h post infection at a MOI 1 (Figure 2A). The resistant cell lines B57 and A2 did not produce any virus (Figure 2A). In contrast, the highly susceptible A22 cell line showed a strong virus production. The sensitive A3 cell line also supported a strong increase of the virus titer between 48 and 96 hours post-infection in accordance with their susceptible phenotype, while B45 and A36 lines presented an intermediary phenotype, with a delayed and lower increase in virus titer. The RTG cell line that has been kept in culture for a long time - more than 100 passages - shows a high virus production, as expected from previous observations.

To further characterize the early steps of viral infection in the different cell lines, the amount of viral N transcripts was quantified using real time RT-PCR analysis at 4 hours post-infection (MOI 1) (Figure 2B). Since the gene encoding the N protein is the first and the most expressed from the VHSV genome, these experiments provided a rough assessment of the efficiency of the first steps of the virus replication cycle. As shown in figure 2B, we observed different phenotypes well consistent with the pattern of virus production (Figure 2A) and the CPE shown in Figure 1. While A3 and A22 cell lines presented significant amounts of N transcripts as early as 4 hours post infection, very low levels were detected in B57 and A2 resistant cell lines at this time. Intermediate values were found for cell lines previously mentioned as moderately susceptible to the virus. Unexpectedly, the expression of N transcript in the susceptible RTG cells was still rather low at 4 hours post infection, while the virus production is very high at later time points.

Finally, the viral N protein can only be detected in the most susceptible clones (A22 and A3) and one intermediary (B45) cell lines by Western blot analysis. In contrast, we were not able to detect viral N protein expression in the resistant clones (A2 and B57) even upon longer time of exposure (data not shown). Protein loading control was performed by red ponceau staining of the nitrocellulose membrane (Figure S1). While no protein can be detected after 1 hour of virus absorption, a drastic difference was observed between resistant and susceptible cells after 48 hours of infection. This result was in good accordance with the amount of N mRNA measured 4 hours post infection (Figure 2C).

Thus, the assessments of virus infection at the different stages were well correlated, either at the early time points through viral gene transcription analysis or later during the viral cell cycle (CPE and viral production). These observations suggested that the differences of susceptibility between cell lines are determined at least in part during the first steps of the infection.

### Cell line susceptibility to the virus infection mirrors susceptibility of parental fish clones

**Figure 3:**
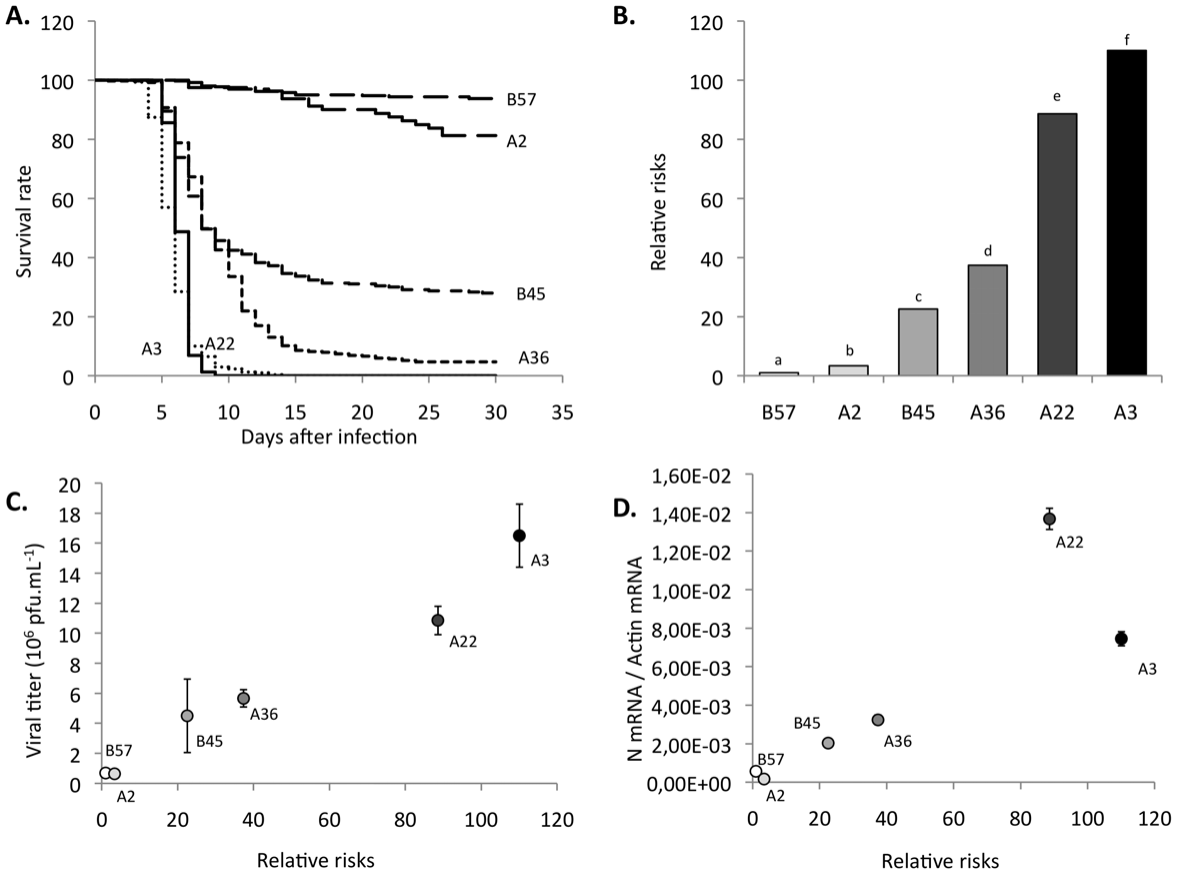
The susceptibilities of fish clones and cell lines to VHSV infection are highly correlated. (A) Kaplan-Meier estimation of survival function for every fish clone. This estimation is calculated from the 9 independent waterborne infections with VHSV corresponding to 5166 infected fish (including data from (49)). (B) Relative risks relative to the B57 clone (R = 1). Relative risks are estimated from the same dataset as above using the “Survival kit” software. The program also computes a chi^2^ test between each clone pair. Letters above each column design a clone significantly different from all the others. All paired tests indicated statistically significant differences between clones. (C) Correlation between risks of fish clones and viral production 96 hours post infection. Linear regression: R = 0.99. (D) Correlation between risks of fish clones and N viral gene expression 4 hours post infection in cell lines. Linear regression: R = 0.85.

The mortality of the parental fish clones on day 30 post-infection was highly variable and revealed a vast diversity of resistance levels to the VHSV infection [21]. Susceptibility of the derived cell lines appeared to be in global accordance with the mortality rates of the parental fish clones after bath viral infection [21], and the most resistant cell lines A2 and B57 were derived from the fish clones with the lowest mortality. However, final mortality is only a rough indicator of the fish susceptibility, and we therefore calculated a quantitative index by performing a longitudinal statistical analysis of the kinetics of fish mortality. Using the “Survival kit” software [60], we first estimated the raw survival function of each clone (Figure 3A). A statistical analysis of the kinetics of mortality based on a simple Cox proportional hazards model was performed. Choosing the most resistant clone (*i.e.* B57) as a reference, the different fish clones could be ranked according to their relative risk defined as the exponential of the estimate of the clone effect in the Cox model (Figure 3B). Ability of the respective derived cell lines to produce virus 96 h post-infection appeared to be highly representative of the relative risk of the fish clone from which they originated. Both viral production (Figure 3C) and N mRNA level (Figure 3D) were remarkably correlated to the *in vivo* clones susceptibility (coefficient of correlation was 0.99 and 0.85 respectively). This excluded that a systemic immune response would be the main factor determining the resistance level of fish clones. Similar phenotypes were observed with additional cell lines produced from other fish individuals of the same clones (data not shown). Thus, the susceptibility of the fibroblasts is highly representative of the susceptibility of the fish from the same genetic background, which indicates that the resistance is based on intrinsic or innate mechanisms still acting in the isolated fibroblastic cell lines.

This correlation was consistent with virus titration on fin explant cultures (Figure S2). As for the fibroblastic cell lines, the virus production on fins kept in culture *in vitro* was high for susceptible A3 and A22 and lower for B57, A2, as well as for B45 and A36. These results showed that the cell response was not affected by the culture process and confirmed the good correlation between viral growth on fin explants and fish survival described in [59].

### A2 and B57 cell lines express different resistance mechanisms

**Figure 4:**
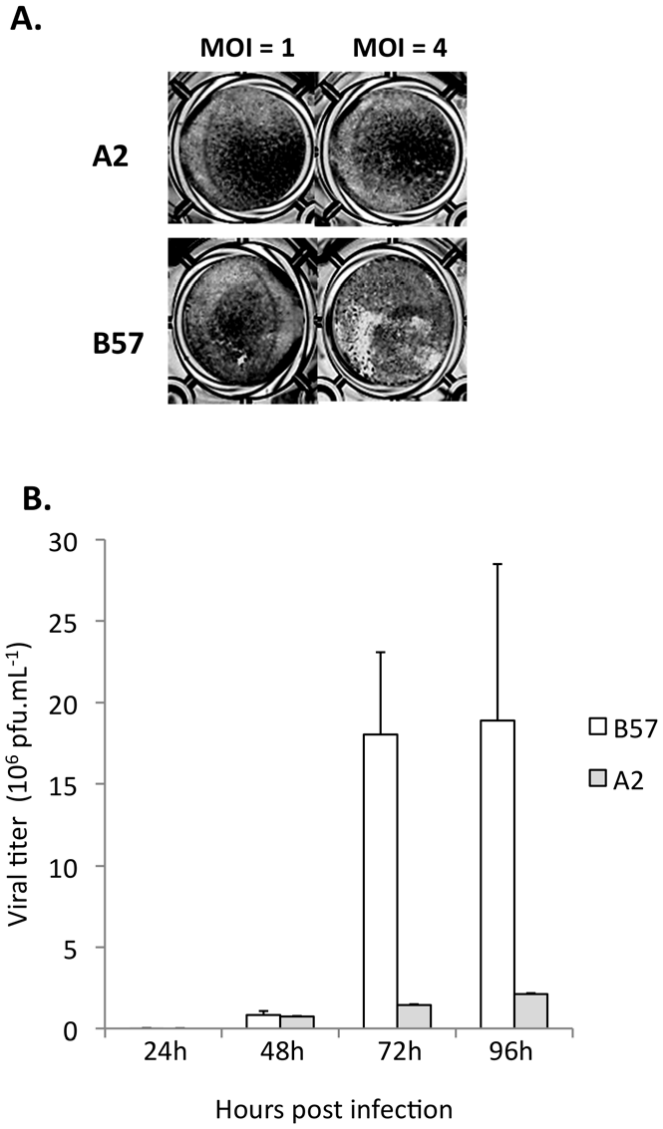
Different resistant phenotypes of cell lines A2 and B57. (A): viral production in cells infected by VHSV at MOI 4. Two independent infections were performed. Viral titer was estimated by plaque assay on EPC cells in duplicates. (B): CPE after VHSV infection at MOI 1 and MOI 4. Cells were fixed 3 days post infection and colored with crystal violet.

Cell lines from the two resistant backgrounds (A2 and B57) constitute interesting models to dissect the genetic basis of the resistance mechanisms. Several lines of evidence suggested that the mechanisms responsible for the resistance to the virus were different in A2 and B57: (1) the final survival rate and the kinetics of fish mortality were significantly different for A2 and B57 as shown by the relative risk produced by the survival analysis (2) VHSV could be re-isolated from around 10% of the B57 infected fish (even if they showed no clinical sign) while it was never the case from the A2 animals ([21] & data not shown) (3) whereas no sign of infection was ever observed on A2 cell monolayer, a few plaques were present at high MOI for B57 cells (Figure 1A). To further compare the resistance of A2 and B57 cell lines to VHSV, we infected sub-confluent monolayers with the virus at MOI 1 and MOI 4. We observed no CPE on A2 cell line even at MOI 4, while the B57 monolayer was partially destroyed 72 h post infection at MOI 4, but not at MOI 1 (Figure 4A). Accordingly, the viral production was minimal for both clones when infected at MOI 1 (as shown in Figure 2A). The viral titer in the supernatant of A2 and B57 infected at MOI 4 was determined at 24, 48, 72 and 96 hours post-infection (Figure 4B) and revealed a significant difference between the two lines: while the virus titer remained low (<1 10^6^ pfu/ml) for both cell lines during the first two days of infection, it reached 1.7 10^7^ pfu/ml at 72 hours for B57 while it was increasing slowly in A2 supernatant and was still below 3 10^6^ pfu/ml at 96 h post infection. In comparison, a titer over 10^8^ pfu/ml was observed with the highly susceptible cell line A22 in the same conditions. These observations were consistent with the CPE observed on monolayers, and indicated that the mechanisms responsible for the high resistance of A2 and B57 cells to VHSV infection are different, qualitatively or quantitatively.

### Early expression of IFNφ after VHSV infection only by the resistant cell line B57

**Figure 5:**
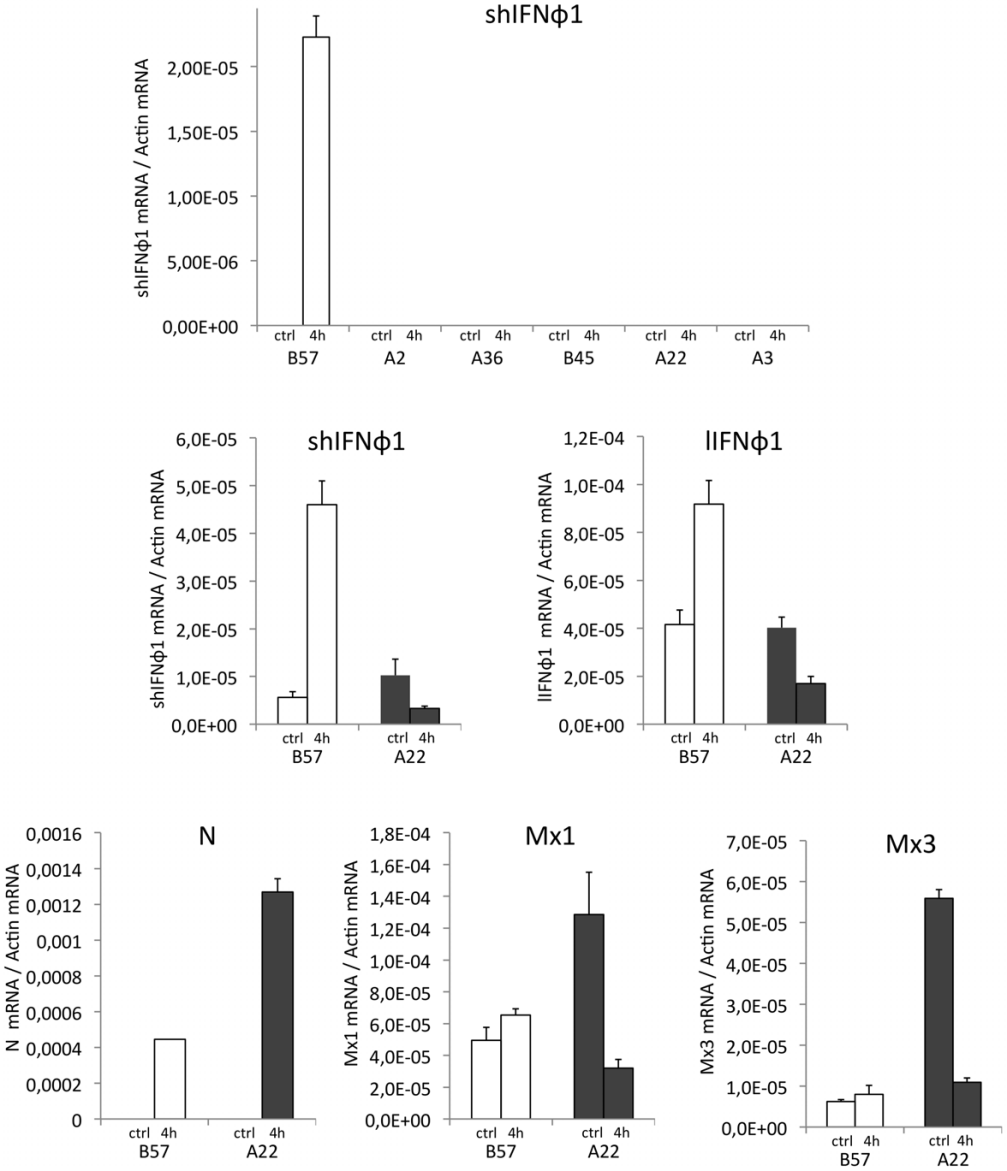
The resistant phenotype of B57 cells results from combined early IFNφ1 and intrinsic immunity. (A): Expression of the functional IFNφ1 (shIFNφ1) in cell lines. Gene expression was measured by qPCR 4 hours post infection by VHSV (MOI 1) or in mock infected cells (ctrl). shIFNφ1 transcript copy numbers were normalized on the ß-actin expression (measured ratio of mRNA of interest/ß-actin mRNA). The mean of three experiments is shown. (B): Expression of IFN φ1, MxI and Mx3 interferon induced genes and viral N mRNA in B57 and A22 cell lines. Expression was measured by qPCR 4 hours post infection or in control cells. Higher amount of template was used, allowing detection of the basic expression levels of the different genes. Primers used are presented in Table 1.

IFNφ (also known as type I IFN) is the central mediator of the fish innate antiviral response and provided an obvious candidate for the explanation of the diversity of resistance among clones. To check that the IFNφ system was functional and supported antiviral activity in the cell lines, we verified that incubation with poly I∶C prior to virus infection induced protection of cell monolayers in a dose-dependant manner, except for the A2 clone that never showed any CPE (data not shown). Since the level of N mRNA expression 4 hours post infection already mirrored the susceptibility level of cell lines, early mechanisms should be involved. To look for a relevant innate antiviral response participating to the resistance to VHSV, we therefore analyzed the early induction of IFNφ by the virus infection. In fish, the induction of IFNφ involves alternative splicing and promoter usage: in non-infected cells, the first in-frame AUG codon is located downstream of the leader peptide and the constitutive IFN mRNA therefore encodes a non-secreted IFN devoid of leader peptide. Upon viral infection, another promoter is used downstream, and five exons are spliced together to yield a shorter mRNA encoding a functional secreted protein. This additional regulation was first described in zebrafish [51] but has been later found in rainbow trout IFNφ1 [20]. We therefore quantified the short IFNφ 1 (shIFNφ1) transcript encoding a functional cytokine 4 hours post infection in the cell lines (Figure 5A). At this early time point, we found a significant amount of shIFNφ1 transcripts only in the B57 cell line, suggesting that the IFN system is involved in the VHSV resistant phenotype of this particular genetic background. In contrast, no shIFNφ1 mRNA could be detected in the other resistant A2 cell line, or in the susceptible cell lines. At this point, it was important to check that the effector pathways downstream of IFNφ were effective against the viral infection. To do so, we incubated cell lines with serial dilutions of poly I∶C overnight before infection, and we observed the cytopathic effect induced by the virus (Figure S3A). Our results indicate that poly I∶C treatment is indeed inducing a full protection of susceptible cells at concentrations higher than 10 µg/ml, confirming that at least part of the IFN pathway was effective downstream IFN. At higher dilutions, the destruction of the monolayer depended on the genetic background and was consistent with the susceptibility of cell lines previously described (Figure 1). Additionally, when the birnavirus Infectious Pancreatic Necrosis Virus was used in a similar experiment (Figure S3B), the cell lines A3, A36 and A22 appeared much more susceptible than B57 and A2 like after VHSV infection, suggesting that a general antiviral mechanism is involved. In contrast, the B45 cell line was fully resistant, which was indicative of other IPNV-specific mechanisms. All cell lines were protected after incubation with higher doses of poly I∶C. Taken together, these results show that poly I∶C treatment induces a protection against viruses from different families in most cells lines, which is consistent with an implication of IFN.

### The resistant phenotype of B57 cells is partly based on intrinsic antiviral mechanisms

We further investigated the role of IFNφ in the B57 resistant phenotype, using one of the most susceptible clones (A22) as a control for comparison. Both shIFNφ-1 and mRNA encoding the long isoform (lIFNφ-1) were both clearly induced in B57 cells 4 hours post infection (MOI 1). Interestingly, the induction of shIFNφ-1 mRNA was stronger than for lIFNφ-1, as observed in the zebrafish model [51]. Both IFN transcripts were down regulated in A22 cells, probably reflecting the virus induced shut-off of cellular RNA synthesis. The viral N transcript was much more expressed in A22 compared to B57 cells 4 hours post infection (MOI 1). To determine if this early difference could be due to effectors induced by IFN, we assessed the expression level of two typical IFN-induced antiviral genes, Mx1 and Mx3. While IFNφ1 is already up-regulated 4 hours post inoculation, we did not observe Mx1 or Mx3 induction in B57 cells. Hence, a significant viral inhibition was observed in B57 cells shortly after infection when IFN-induced effector genes were still absent, suggesting the implication of an (early) intrinsic antiviral activity in addition to interferon-dependent mechanisms. In fact, both Mx1 and Mx3 were expressed in the non-infected B57 and A22 cells at low, slightly different levels (Figure 5B). Upon infection, both Mx1 and Mx3 mRNAs showed a down-regulation in A22 cells 4 hours post infection, as observed for IFNs. Mx genes are induced later, after expression of IFN (data not shown).

**Figure 6:**
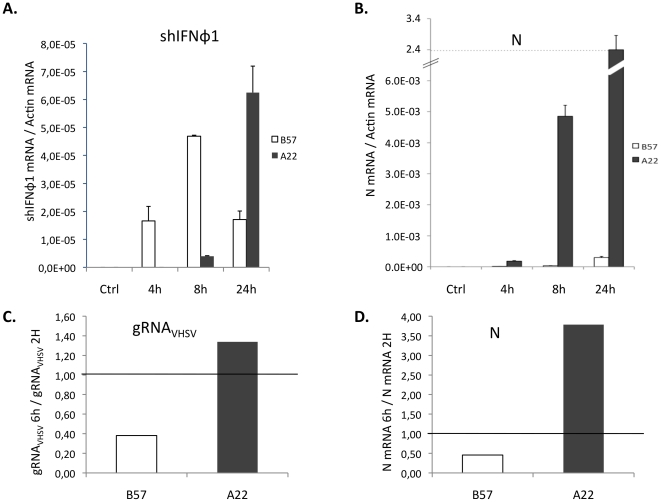
Early IFN- dependent and independent viral inhibition in B57 cells. (A): The expression of shIFNφ1 transcript in A22 and B57 cells after VHSV infection was measured by qPCR 4, 8 and 24 hours post infection by VHSV or in mock infected cells (ctrl). mRNA copy numbers were normalized on ß-actin expression (measured ratio of shIFNφ1 mRNA/actin mRNA). The mean of three experiments is shown. (B): Expression of N viral gene in cells after VHSV 07-71 infection at different time. Expression of gene is measured by qPCR after 4, 8 and 24 hours of infection by VHSV 07-71 or without viral infection (ctrl). Gene expression is evaluated relative to ß-actin expression (measured ratio of VHSV N mRNA/actin mRNA). The mean of three experiments is shown. (C): Viral genomic RNA (strand+ plus strand−) was quantified using qPCR. The virus replication was assessed by the ratio of virus genome at 6 h versus 2 h post-inoculation. (D): The mRNA encoding the viral N protein was quantified in parallel and the ratio N mRNA 6 hours post inoculation/N mRNA 2 hours post inoculation is represented.

To check that the induction of IFN expression by the virus was delayed in A22 compared to B57, we measured shIFNφ-1 transcripts 4, 8 and 24 hours post infection (Figure 6). When cells were infected at MOI 1, the N mRNA reached a very high expression rate in A22 cells - more than twice the actin mRNA level at 24 hours post infection– and the shIFNφ1 was finally strongly up-regulated following the considerable virus production in these cells (Figure 6A). In contrast, the expression level of shIFNφ1 mRNA decreased at 24 hours post infection in B57 cells (Figure 6A). At this stage, no CPE was observed, and the virus N transcript was expressed at a detectable but low level (Figure 6B) in B57 cells. Hence, the resistance of the B57 cells appears to be firstly due to an intrinsic component, in addition to the early induction of IFN.

To investigate the impact of this factor on the early replication of the virus, we quantified the viral genomic RNA in B57 and A22 cell lines using RT-QPCR primers located in two different ORF. Cells were incubated with the inoculum until harvesting to avoid any possible bias due to medium change after the adsorption step. We consistently observed that the total amount of genomic viral RNA (strand+ plus strand−) decreased in B57 cells and increased in A22 cells between 2 and 6 hours post infection, suggesting that an early mechanism hampering viral replication arose in B57 cells (Figure 6C). Accordingly, the expression level of the N mRNA followed the same trend (Figure 6D). In good accordance with the results shown above, we observed an up-regulation of the shIFNφ1 induction only in B57 cells 6 hours post inoculation, but not change compared to the control in any cell line, 2 hours post inoculation (data not shown).

## Discussion

In this work, we showed a remarkable correlation between the *in vivo* susceptibility to VHSV infection of isogenic clones of rainbow trout and the susceptibility of the cell lines derived from these animals. Our results demonstrated that the variation of susceptibility to the virus depends on the genetic background and that the major pathways responsible for resistance are independent of the specific immune response. We could establish that the resistant phenotypes are based on multiple precocious mechanisms, including the capacity to mount an early IFNφ response in some cases.

We directly demonstrated that the genetic basis for the resistance to the virus does not rely on the specific response and is most probably not linked to the MHC: the tight correlation of resistance between fish clones and their derived fibroblast-like cell lines points up the contribution of innate/intrinsic mechanisms. A strong association has been demonstrated between MHC haplotypes and the resistance to several viral infections including AIDS in human, Newcastle disease in poultry and ISA in Atlantic salmon, suggesting that T cell mediated immunity was involved. In rainbow trout, the susceptibility of fish families to VHSV was recently correlated to the expression of genes involved in adaptive immunity [62], which may be linked to a T-cell dependent synthesis of neutralizing antibodies [56], [63]. However, several observations indicated that the resistance to waterborne rhabdovirus infection may rely on the innate immune response, in accordance with our results. Thus, Purcell and colleagues [64] described an association between the early viral load and the level of resistance to IHNV in young rainbow trout families, suggesting that early antiviral mechanisms are pivotal for the host survival. In the same line, fish survival to VHSV waterborne infection was strongly correlated to the viral replication on excised fin tissue 24 h after *ex vivo* infection [59], which was confirmed in this study with susceptible and resistant fish clones. This virus production assay on fin culture could even predict the susceptibility of the progeny of individual fish [59]. As fins have been shown to constitute the major entry point of the virus [65], the control of virus spreading in the early steps of infection appeared to explain partly the genetic resistance to the virus.

The susceptibility level of fibroblast-like cell lines could be determined by an innate antiviral response inducible by the infection, or by intrinsic cellular factors modulating the innate response or directly the viral cycle (entry, replication, assembly…). As in mammals, the fish innate antiviral response is orchestrated by typical virus-induced IFNs - also known as IFNφ - with antiviral activity [46], [49], [52], [66], [67] and many interferon stimulated genes (ISGs) that are either conserved in all vertebrates (*ISG15, viperin, Mx*,…) [68], [69]–[71] or specific to teleost fish (*fintrim, vig2*) [72], [73]. Pre-stimulation with poly I∶C indicated that the IFN system of each cell line was functional, since a certain dose of poly I∶C could induce cell protection. Yet, this assay did not discriminate the impact of differential IFN induction and intrinsic cell factors. We therefore analyzed the early IFNφ1 induction, since a key factor for the fate of the infection is the kinetics of IFN production. Only the resistant cell line B57 showed an early IFNφ1 response, indicating that the mechanisms involved were different in B57 and in the other resistant cell line A2. In fact, a large repertoire of IFNφ has been discovered in Atlantic salmon [74], and the rainbow trout IFNφ diversity is rapidly expanding [66]. As the trout genome sequencing is still ongoing, the final trout IFNφ repertoire is not available and we could not directly rule out that A2 cells constitutively expressed IFN genes unknown so far. However, we did not detect any antiviral activity in the supernatant of A2 cells used in a plaque assay, further suggesting that these cells do not resist the virus infection through IFN production. In spite of the higher resistance of A2 cell line to the infection at high MOI compared to B57 and of the absence of virus in survivor fish, the A2 fish are significantly less resistant to the viral infection than B57 fish, suggesting that additional factors are important at the organism level. Interestingly, A2 fish are the most resistant of all clones to the infection by another fish rhabdovirus, IHNV. This higher resistance is especially marked to infections of fry weighing less than one gram, since B57 are still poorly resistant at this stage [21]. As fibronectin was shown to mediate the entry of both VHSV and IHNV in fish fibroblasts [75], it is tempting to speculate that an impaired virus entry in A2 cells may be involved in their resistance to rhabdovirus infection. The capacity of viruses to infect other cell types *in vivo* could modulate the resistance level at the organism level.

A significant contribution of early IFN response in the B57 phenotype was suggested by the diversity of viruses to which B57 shows a robust resistance: in addition to VHSV, the B57 cell line was resistant to IHNV and to a birnavirus, the Infectious Pancreatic Necrosis Virus (IPNV) (data not shown). Also, the B57 fish clone was one of the most resistant clone to the fish orthomyxovirus-like virus causing the infectious salmon anaemia, ISAV [19]. Taken together, these observations suggest that the B57 resistance involves a general mechanism inhibiting a wide range of viruses, which is very suggestive of an implication of the IFN system. In fact, the level of IFN expression was already decreasing 24 hours post infection in B57 cells, when the virus had not succeeded to start a successful infection. Interestingly, the production of the viral transcript encoding the N protein was strongly impaired 4 hours post infection (MOI 1) in B57 cells, when the expression of effector ISGs such as Mx genes was still undetectable. Hence, the difference of resistance at this time could not be explained by the IFN response and ISGs, but rather by other cellular restriction mechanisms. Thus, the basis of the B57 resistance to viral infections appears to proceed from a combination of early IFN induction and intrinsic cell restriction mechanisms. The decrease of the amount of viral genomic RNA between 2 and 6 hours post infection in resistant B57 cells - in contrast to the susceptible A22 cells – suggest that the early viral replication is hampered in B57. We can imagine that this double line of defense contributes to the very strong level of resistance of this clone after *in vivo* infection.

Our results also showed differences of response between susceptible clones. Mortality kinetics during the infectious challenge were significantly different as shown by the relative risk. Differential cellular responses were observed both in term of viral production and CPE: for example, B45 cell monolayers were quickly destroyed by the infection at MOI 1, in spite of a viral production much lower than with A22 or A3 cells that was in agreement with the intermediate survival rate of fish after waterborne challenge. The A36 cell line was fairly susceptible to the VHSV infection, and A36 fish clones were susceptible to both VHSV and IHNV [21]. In contrast, this fish clone was among the most resistant to ISAV infection [19]. While B57 fish did resist the ISAV infection without clinical signs, A36 fish showed very clear exophthalmia, a swollen belly and lateral petechiae but generally survived, possibly through systemic antiviral mechanisms. The A36 cell line was also quite resistant to the birnavirus IPNV, suggesting that the responses triggered by different viruses were of different efficacies.

In conclusion, we have established a dual collection of double haploid fish clones and isogenic derived fibroblast-like cell lines spanning a wide range of susceptibility to VHSV infection. Cell lines susceptibility to the virus mirrors the susceptibility of parental fish clones, emphasizing the role of antiviral innate/intrinsic mechanisms. Thus, resistance and susceptibility to viruses involve multiple mechanisms, in addition to the interferon response. Progress in sequencing technologies and genomics promises fast advances for the coming years, and further studies may reveal that new major genes confer a predisposition to many common diseases in different species from fish to human.

## Supporting Information

Figure S1 **Normalization of protein loading in the analysis of the NSHV expression.** Normalized amounts of each cell lysates (50 µg of proteins/well) were analyzed by SDS PAGE. To evaIuate the protein loading, nitrocellulose membrane was stained with Red ponceau in absence of available antibody directed against trout proteins. Lines: 1 hour: cells were incubated with VHSV 07-71 during only one hour and lysates prepared for western blotting. (1): B57 (2): A2 (3): B45 (4): A22 (5): A3 (6): RTG. 48 hours: cells were infected as described in Material and Methods, and lysates prepared 48 hours post infection for western blotting. (7): B57 (8): A2 (9): B45 (10): A22 (11): A3 (12): RTG. Ctrl: EPC cells transfected with NSHV cDNA.
(TIFF)

Figure S2 **Viral titer in fin explants after 4 days of infection by VHSH 07-71.** Fin explants were infected as indicated in Material and Methods. Log(viral titer) are expressed in pfu.mL^−1^ per mg of tissue. The two culture experiments must be considered independently and viral titers cannot be directly compared since the inoculum cannot be properly normalized in this protocol.
(TIFF)

Figure S3 **IFN Induction by Poly I∶C protects cell monolayers against two different viral infections in a dose-dependant manner.** Cells were treated with increasing concentrations of Poly I∶C overnight before infection. Poly I∶C concentrations are in µg/mL. Cells were infected and kept 3 days with the virus inoculum, then fixed and colored with crystal violet. Monolayer destruction 3 days post infection by VHSV (MOI 1) (A) or by IPNV (MOI 1) (B). **V:** Cell monolayer infection without Poly I∶C pre-treatment. **Ctrl:** Non-infected cell monolayers without Poly I∶C pre-treatment.
(TIFF)
