# Supplementary figures and images for: Genetic Resistance to Rhabdovirus Infection in Teleost Fish Is Paralleled to the Derived Cell Resistance Status
Source: PLoS One. 2012 Apr 13;7(4):e33935. doi: 10.1371/journal.pone.0033935 (PMC3326022; doi:10.1371/journal.pone.0033935)

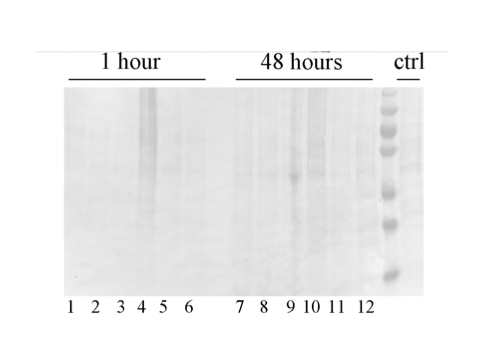

Supplement: Figure S1 — Normalization of protein loading in the analysis of the NSHV expression. Normalized amounts of each cell lysates (50 µg of proteins/well) were analyzed by SDS PAGE. To evaIuate the protein loading, nitrocellulose membrane was stained with Red ponceau in absence of available antibody directed against trout proteins. Lines: 1 hour: cells were incubated with VHSV 07-71 during only one hour and lysates prepared for western blotting. (1): B57 (2): A2 (3): B45 (4): A22 (5): A3 (6): RTG. 48 hours: cells were infected as described in Material and Methods, and lysates prepared 48 hours post infection for western blotting. (7): B57 (8): A2 (9): B45 (10): A22 (11): A3 (12): RTG. Ctrl: EPC cells transfected with NSHV cDNA. (TIFF) [file pone.0033935.s001.tiff]

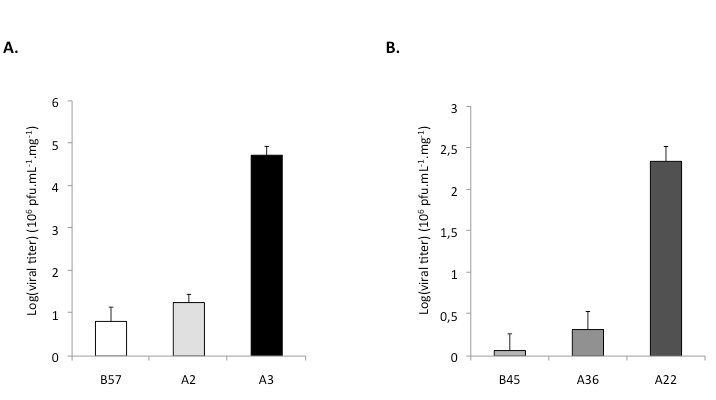

Supplement: Figure S2 — Viral titer in fin explants after 4 days of infection by VHSH 07-71. Fin explants were infected as indicated in Material and Methods. Log(viral titer) are expressed in pfu.mL−1 per mg of tissue. The two culture experiments must be considered independently and viral titers cannot be directly compared since the inoculum cannot be properly normalized in this protocol. (TIFF) [file pone.0033935.s002.tiff]

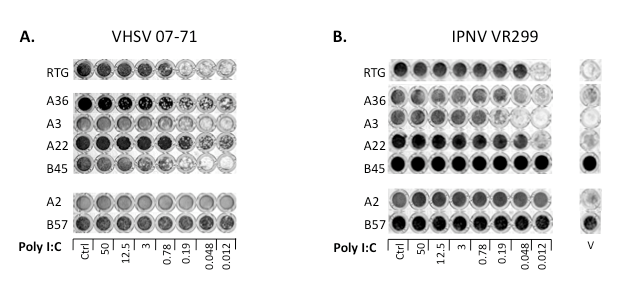

Supplement: Figure S3 — IFN Induction by Poly I∶C protects cell monolayers against two different viral infections in a dose-dependant manner. Cells were treated with increasing concentrations of Poly I∶C overnight before infection. Poly I∶C concentrations are in µg/mL. Cells were infected and kept 3 days with the virus inoculum, then fixed and colored with crystal violet. Monolayer destruction 3 days post infection by VHSV (MOI 1) (A) or by IPNV (MOI 1) (B). V: Cell monolayer infection without Poly I∶C pre-treatment. Ctrl: Non-infected cell monolayers without Poly I∶C pre-treatment. (TIFF) [file pone.0033935.s003.tiff]
